# Impact of Elevated CO_2_ on Tobacco Caterpillar, *Spodoptera litura* on Peanut, *Arachis hypogea*

**DOI:** 10.1673/031.012.10301

**Published:** 2012-08-27

**Authors:** M Srinivasa Rao, D Manimanjari, M Vanaja, CA Rama Rao, K Srinivas, Vum Rao, B Venkateswarlu

**Affiliations:** ^1^ Division of Crop Sciences, Central Research Institute for Dryland Agriculture, Hyderabad, 500059, India; ^2^ Section of Design and Analysis, Central Research Institute for Dryland Agriculture, Hyderabad, 500059, India; ^3^ Division of Resource Management, Central Research Institute for Dryland Agriculture, Hyderabad, 500059, India; ^4^ All India Coordianted Research Project on Agrometerology (AICRPM), Central Research Institute for Dryland Agriculture, Hyderabad, 500059, India; ^5^ Director, Central Research Institute for Dryland Agriculture, Hyderabad, 500059, India

**Keywords:** atmospheric carbon dioxide, consumption, foliage, insect performance indices

## Abstract

If the carbon dioxide (CO_2_) concentration in the atmosphere changes in the future, as predicted, it could influence crops and insect pests. The growth and development of the tobacco caterpillar, *Spodoptera litura* (Fabricius) (Noctuidae: Lepidoptera), reared on peanut (*Arachis hypogea* L.) foliage grown under elevated CO_2_ (550 ppm and 700 ppm) concentrations in open top chambers at Central Research Institute for Dryland Agriculture, Hyderabad, India, were examined in this study. Significantly lower leaf nitrogen, higher carbon, higher relative proportion of carbon to nitrogen and higher polyphenols content expressed in terms of tannic acid equivalents were observed in the peanut foliage grown under elevated CO_2_ levels. Substantial influence of elevated CO_2_ on *S. litura* was noticed, such as longer larval duration, higher larval weights, and increased consumption of peanut foliage by *S. litura* larvae under elevated CO_2_ compared with ambient CO_2_. Relative consumption rate was significantly higher for *S. litura* larva fed plants grown at 550 and 700 ppm than for larvae fed plants grown at ambient condition. Decreased efficiency of conversion of ingested food, decreased efficiency of conversion of digested food, and decreased relative growth rate of larvae was observed under elevated CO_2_. The present results indicate that elevated CO_2_ levels altered the quality of the peanut foliage, resulting in higher consumption, lower digestive efficiency, slower growth, and longer time to pupation (one day more than ambient).

## Introduction

The fourth assessment report of the InterGovernmental Panel on Climate Change (2007) reconfirmed that the atmospheric concentrations of carbon dioxide (CO_2_), methane, and nitrous oxide greenhouse gases have increased markedly since 1750. The report showed that these increases in greenhouse gases have resulted in warming of the climate system by 0.74° C over the past 100 years. The projected increase in temperature by 2100 was set at 1.8 to 4.0° C. Atmospheric concentrations of CO_2_ have been steadily rising, from approximately 315 ppm in 1959 to a current atmosphere average of approximately 385 ppm. Even if the annual flow of emissions did not increase beyond today's rate, the stock of green house gases in the atmosphere would reach double pre-industrial levels, or 550 ppm CO_2_, by 2050, and would continue growing thereafter (Stern 2007). This increase is likely to affect biota indirectly via climate change, and directly by producing changes not only in plant growth and allocation, but also in plant tissue chemical composition. Legumes, being more responsive to elevated CO_2_ than other plants, will have a competitive advantage (Ainsworth and Long 2005) when grown under elevated CO_2_. Within legume species, nodulating genotypes, or N fixers, are more responsive to elevated CO_2_ than non-fixers (Ainsworth et al. 2004).

Many plant species respond to enriched atmospheric CO_2_ by enhanced photosynthetic rates and increase in biomass, as well as alterations in leaf quality factors. Insect and host plant interactions change in response to the effects of CO_2_ on nutritional quality and secondary metabolites of the host plants. In atmospheres experimentally enriched with CO_2_, the nutritional quality of leaves declined substantially due to dilution of nitrogen (N) by 10–30% Coley and Markham 1998). Lower foliar N content due to elevated CO_2_ causes an increase in food consumption by herbivores by up to 40% (Hunter 2001). Many species of herbivorous insects will confront less nutritious host plants under elevated CO_2_, which may induce both lengthened larval developmental times and greater larval mortality (Coviella and Trumble 1999). Increased levels of CO_2_ will enhance plant growth, but may also increase the damage caused by some phytophagous insects (Gregory et al. 2009). Reduced leaf N, and consequent compensatory feeding, is less likely in legumes, which may overcome N limitation by increased nodulation and N fixation. However, increased folivory in soybean, (Rogers et al. 2006) and increased damage by herbivores in some legume species, despite avoiding the N dilution, has been reported (Zavala et al. 2009).

Peanut (*Arachis hypogaea* L.), also known as groundnut, earthnut, and ground bean, is the world's fourth most important source of edible vegetable oil, and third most important source of vegetable protein. Peanut was grown on 23.91 million hectares worldwide, with a total production of 36.60 million tons, and an average yield of 1531 kg/hectare in 2009 (http://faostat.fao.org). China, India, Nigeria, the United States of America, and Myanmar are the major peanut growing countries. India is the second largest producer of peanut in the world, with an average annual production of 5.51 million tons (http://faostat.fao.org). Developing countries in Asia, Africa, and South America account for over 97% of world peanut area, and 95% of total peanut production. Production is concentrated in Asia (50% of global area, and 64% of global production) and Africa (46% of global area and 28% of global production), where the crop is grown mostly by smallholder farmers under rainfed conditions with limited inputs.

Peanut has traditionally been used as a source of oil; however, its worldwide annual protein harvest has reached nearly 4.5 million tons. Crude protein content of whole seed peanuts is estimated to be around 25%, followed by carbohydrates (16%), and monosaturated fats (24%) (http://ndb.nal.usda.gov/ndb/foods/show/4779). Elevated CO_2_ was reported to cause significant increase in total biomass at the final harvest of peanut crop, but decreased final seed yield in selected cultivars (Bannayan et al. 2009).

The peanut crop is attacked by many species of insects that cause damage ranging from incidental feeding to near total plant destruction and yield loss (Wightman and Ranga Rao 1994). Among the damaging species, the tobacco armyworm, *Spodoptera litura* (Fab.), is as a major pest, and can cause yield losses of 35–55%. Larvae feed gregariously on leaves, causing severe defoliation, leaving midrib veins only. Response of herbivory to elevated CO_2_ is highly complex, and the interactions between legumes and insect-herbivores are unclear. The present study was aimed to elucidate the insect-herbivore (*S. litura*) and plant (peanut) interactions under elevated CO_2_.

## Materials and Methods

### Open Top Chamber

Three square type open top chambers (OTCs) of 4×4×4 m dimensions were constructed at the Central Research Institute for Dryland Agriculture, Hyderabad (17.38° N; 78.47° E). Two were for maintaining elevated CO_2_ concentrations of 550 ± 25 ppm CO_2_ and 700 ± 25 ppm CO_2_, and one was for ambient CO_2_ (380 ppm CO_2_). Carbon dioxide gas was supplied to the chambers, and maintained at set levels using manifold gas regulators, pressure pipelines, solenoid valves, rotameters, sampler, pump, CO_2_ analyzer, PC linked Program Logic Control, and Supervisory Control and Data Acquisition. The fully automatic control and monitoring system, which included the CO_2_ analyzer, PLC, and SCADA program for PC, enabled maintaining the desired level of CO_2_ within the OTC's, as well as maintaining temperature and relative humidity. The system monitored continuously the concentration of CO_2_, temperature, and relative humidity within the OTCs. The uniformity of the CO_2_ was maintained by pumping CO_2_ gas diluted with air by an air compressor. The air was sampled from the center point of the chamber through a coiled copper tube that could be adjusted to different heights as the crop grew. The equipment was monitored, but controlling the CO_2_ in the OTCs was fully automatic, and the desired CO_2_ level was maintained throughout the experimental period. Peanut (variety JL-24) seeds were sown in June 2011 in the three OTCs, and crop plants were maintained during the entire crop season until December 2011.

### Feeding trials

The egg masses of *S. litura* were collected from a field and maintained at the entomology laboratory of the Central Research Institute for Dryland Agriculture. The cultures were maintained in controlled conditions at 25 ± 2° C, with a 14:10 L:D cycle. Stock cultures were maintained on leaves of peanut plants grown in the open field condition. At 10:00 on the day of initiating the feeding trial, 10 freshly hatched neonates forming one replication were placed in a petridish of 110 mm diameter and 10 mm height. Six replications (60 larvae) were kept for each CO_2_ level, making a total of 180 larvae. Before placing the neonates, a moistened filter paper was kept at the bottom of the petridish to maintain leaf turgidity. Neonate larvae were fed with peanut leaves brought from respective open top chambers at different CO_2_ concentrations. The feeding trial was conducted within a 30 day period, between 30 to 60 days age of peanut plants. Each day, the youngest fully expanded leaves were collected and used for the feeding trial. A weighed quantity of leaf was offered to the larvae. The petridishes were then placed in a controlled chamber maintained at 20° C. After 24 hours, at 10:00 the next day, the petridishes were opened, the weight of the ten larvae together was recorded, and the larvae were placed back in the petridish, after preparing it in the same manner as described earlier, with a new leaf of known weight. The leaf remaining after feeding and fecal matter excreted by the ten larvae was dried to constant weight at 40° C in an oven, and dry weights were recorded. The same process was repeated each day for four days. The weight of the ten larvae was divided by 10 to arrive at mean larval weight. In the same way, mean leaf weight consumed per larva, and fecal matter per larva, were calculated. Statistical analysis was performed using these means. At 10:00 on the fifth day of the trial, each of the 4-day-old larvae was transferred to a transparent plastic jar of 10 cm diameter and 10 cm height in order to prevent congestion and competition among larvae. A moistened filter paper was placed at the bottom of the jar, and a 1-inch strip of moistened filter paper was run around the inner wall of the jar in order to maintain leaf turgidity and air humidity. A more than adequate quantity of leaf material of known weight was placed in the jar, and the 4-dayold larva were placed in their individual jars. The jars were covered with muslin cloth, and kept in the controlled chamber. At 10:00 the next day, each of the jars was opened, the weight of the individual larva was recorded, and the larva was returned to the jar with new moistened filter papers and new leaf material. The leaf remaining after feeding, and fecal matter excreted by the larva, were dried and weighed. This process was repeated daily until the larvae pupated. Although individual larvae were weighed, the weights of the ten larvae derived from each petridish (one replication) were aggregated, and the mean was calculated. Mean leaf weight consumed, and fecal matter per larva, were also calculated similarly. Statistical analysis was performed using these means. After cessation of feeding, pre-pupae were collected and transferred to glass jars. Later, pupae were collected and weighed separately according to the treatments. After the emergence of adults from pupae, the moths were paired, and each pair was kept in a separate plastic container. Adults were fed with 10% honey solution. Egg masses laid were collected separately, and fecundity was estimated.

### Estimation of growth and development indices

The conventional, ratio-based nutritional indices, including approximate digestibility, relative growth (mg.g^-1^ day^-1^), relative consumption rate (mg.g^-1^ day^-1^), efficiency of conversion of ingested food, and efficiency of conversion of digested food were determined gravimetrically following the methods of Waldbauer (1968).

### Biochemical analysis of foliage

Leaf tissue used in the feeding experiments was analyzed for carbon, N (C: N ratio), and polyphenols. To determine carbon and N concentrations, samples were dried at 80° C, and subsequently ground to powder. Leaf carbon and N were measured using a CHN analyzer (Model NA 1500 N, Carlo Erba Strumentazione, Italy) using standard procedures (Jackson 1973). Total soluble polyphenols (hydrolysable tannins, condensed tannins, and non-tannin polyphenols) were determined by the Folin-Denis method (Anderson and Ingramm 1993). For this method, leaf samples were dried at 40° C for 48 hours. Dried leaf samples were ground to powder, and phenolics were extracted with CH_3_OH. The concentration of polyphenols in the extract was determined spectrophotometrically using tannic acid as the standard, and the results were expressed as percentage tannic acid equivalents.

**Figure 1.**
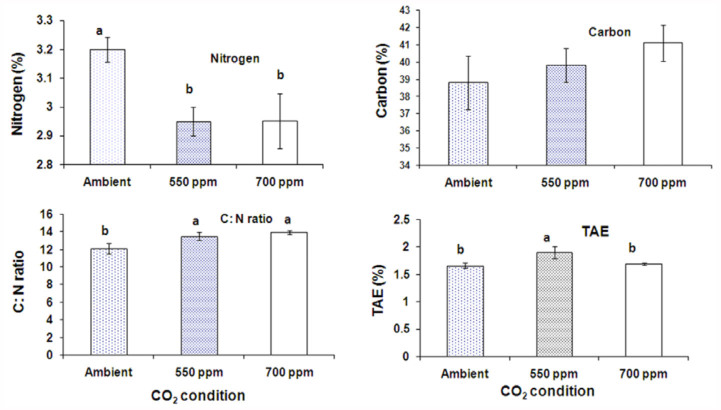
Variation in biochemical constituents of peanut foliage under elevated CO_2_ conditions. High quality figures are available online.

### Statistical analysis

The effects of CO_2_ conditions on larval parameters were analyzed using one- way ANOVA. All treatments were replicated six times (n = 6). Results presented are mean value of each determination (treatment) ± standard deviation. The differences between the mean values of treatments were determined by Tukey's test, and the significance was defined at *p* < 0.05. All statistical analyses were done using SPSS version 16.0.

## Results

### Biochemical analysis of peanut foliage

In this study, leaf N concentration was lower in peanut foliage obtained from elevated CO_2_ conditions than ambient. Nitrogen content was significantly (F_2,4_ = 21.19; *p* < 0.01) lower under elevated CO_2_ conditions (2.95 %) than ambient (3.20 %). In contrast, carbon content was not significantly (F_2, 4_ = 1.78; *p* > 0.05) influenced by CO_2_ condition. (Figure 1). However, the relative C: N ratio was considerably higher (13.5 % and 13.6 %) in elevated CO_2_ peanut foliage than in ambient (12.0%). Polyphenols content measured in terms of tannic acid equivalents was significantly (F_2,4_ = 13.15; *p* < 0.05) higher in peanut foliage under elevated conditions (1.90% and 1.69%) than in ambient (1.66%) (Figure 1).

### Larval growth performance

Relative consumption rate differed significantly among the three CO_2_ treatments (F_5,10_ = 5.41, *p* < 0.05). Relative consumption rate was significantly higher for *S. litura* larva fed plants grown at 550 and 700 ppm than for larvae fed plants grown at ambient condition (by 19% and 24% respectively), but did not differ between larvae fed plants grown at 550 vs. 700 ppm. The similar trend was reflected in the higher consumption (by 55% and 80%, respectively) of peanut foliage by larva (F_5, 10_ = 5.41; *p* < 0.01). The effect of elevated CO_2_ (F_5, 10_ = 3.88, *p* > 0.01) on approximate digestibility of peanut foliage by *S. litura* was not significant across three CO_2_ conditions 550 ppm, 700 ppm, and ambient, despite recording 16–20% higher approximate digestibility under elevated CO_2_ conditions. The effect of elevated CO_2_ on efficiency of conversion of digested food of larvae was significantly (F_5, 10_ = 12.42, *p* < 0.01) lower (by 35% in 550 ppm and 32% in 700 ppm condition) than in ambient. Efficiency of conversion of ingested food for *S. litura* larvae fed on peanut foliage under elevated CO_2_ concentrations was significantly (F_5, 10_ =17.85, *p* < 0.01) reduced (by 25% in both the elevated CO_2_ conditions) than ambient. Relative growth rate of larvae was lower significantly (by 9–10%) when fed on peanut foliage under elevated CO_2_ (F_5, 10_ = 7.97, *p* < 0.01) than ambient (Table 1).

**Table 1.**
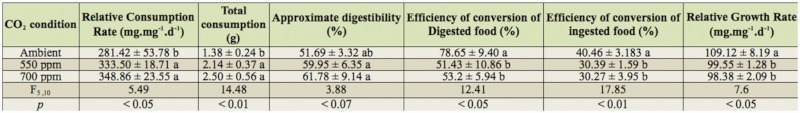
Impact of elevated CO_2_ on larval indices of *Spodoptera litura* on peanut. Means in the same vertical column with different superscripts are significantly different by Tukey's test.

**Table 2.**
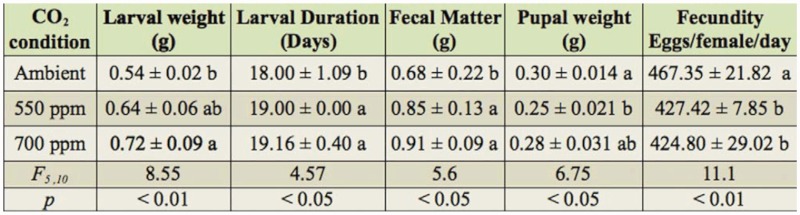
Impact of elevated CO_2_ on growth parameters of *Spodoptera litura* on peanut. Means in the same vertical column with different superscripts are significantly different by Tukey's test.

Larval weights (0.64 ± 0.06 and 0.718 ± 0.09 g) also differed significantly (F_5_, _10_ = 5.41; *p* < 0.01) with elevated CO_2_ condition than ambient CO_2_ (0.541 ± 0.02 g). The higher larval weights were recorded in 550 (18%) and 700 ppm (32 %) CO_2_ conditions. The larval duration was extended significantly under elevated CO_2_ condition than ambient (F_5, 10_ =4.57; *p*<0 .05). The fecal matter release by larvae was also significantly varied and was more in 550 ppm (25%) and 700 ppm (34%) than ambient (F_5, 10_ =5.41; *p* < 0.05). The pupal weights of *S. litura* were lower under elevated CO_2_ conditions than ambient. The less fecund adults were noticed under elevated CO_2_, and resulted in a total of 424– 427 eggs per female per day, as compared with 467 eggs per female per day under ambient (Table 2).

## Discussion

The photosynthesis and growth of many plants are stimulated when plants are grown under elevated CO_2_ condition, and reduction in leaf N content in plants grown at elevated CO_2_, due to faster growth of the plant (Stitt and Krapp 1999), is well known. Biochemical analysis of peanut foliage a significant reduction (8%) of leaf N under elevated CO_2_ conditions as opposed to ambient. It is understood that leaf N content of legumes decreased on average by only 7% under elevated CO_2_, which was less than half the decrease exhibited by the non-legumes C3 plants (Cotrufo et al. 1998). Nitrogen is the single most important limiting resource for phytophagous insects (Mattson 1980), a decrease in foliar N of host plants could affect the development and survival rates of phytophagous insects.

Legumes capable of N fixation are less likely to suffer reduction in N, but may exhibit lower leaf N during early growth stages, as reported by Rogers et al. (2006) in soybean. In this study, biochemical analysis of foliage was done on samples collected from 30-day-old plants. It is possible that N fixation was not fully operational by that time. However, in the absence of specific data on nodulation, N fixation, and biochemical analysis of foliage at later stage of crop growth, no generalization can be made regarding the effect of elevated CO_2_ on foliage N content of the peanut crop.

Increase in atmospheric levels of CO_2_ can cause increases in plant growth rates, and changes in the physical and chemical composition of their tissues (Sudderth 2005). Phytophagous insects are indirectly affected by those changes in their host plants. In the present study, polyphenols were increased in peanut leaves under elevated CO_2_ condition. Increase in phenolics and poly-phenolics concentrations in green leaves under elevated CO_2_ is well known (Agrell et al 2000), and increase in phenolics have a negative influence on the development and fitness of chewing herbivore insects (Yin et al. 2010). In addition, most herbivorous insects appear to be negatively affected by elevated CO_2_ because of the reduction in foliar N and increase in C: N ratio (Bezemer and Jones 1998). In our study, 13% percent increase in C: N ratio was observed under elevated CO_2_ conditions. The reduction in protein content, and increase of C/N ratio in leaves under elevated CO_2_ (Hunter 2001), imply a reduction in food quality that might have caused the higher feeding by larvae. The CO_2_ mediated changes in the peanut foliage (i.e., decreased N and increased polyphenols) in the present study affected the growth and development of *S. litura*, causing higher consumption (54% in 550 ppm and 80% in 700 ppm CO_2_ condition).

The increased larval weight (18% and 32%) with higher fecal matter release was observed under both elevated CO_2_ condition over ambient CO_2_. It was well known that most leaf-chewing insects exhibit compensatory increase in food consumption (Lee et al. 2002). Insects, when fed on elevated CO_2_ grown plants, were shown to increase their individual consumption due to the poor food quality of these plants (Coviella et al. 2000; Hunter 2001). Similarly, the reduction of leaf N content of peanut foliage was noticed, and the feeding trials were conducted during the period of 30–60 days after sowing of the peanut crop. The present results were explained as a response of *S. litura* to reduced foliage quality, especially the variation in N and C: N ratio. Authors observed the similar trend of the effect of elevated CO_2_ on *S. litura* in castor crop (Srinivasa Rao et al. 2009).

In this study, the insect growth performance indices also significantly varied between elevated CO_2_ and ambient conditions. The relative growth rate of larvae fed on elevated CO_2_ peanut foliage was significantly reduced. It is understood that larvae consumed and assimilated more but grew slower (lower relative growth rate), and took one to two days longer of extension to pupation than ambient. Low efficiency of conversion of digested food may result from a requirement of these insects to metabolize digested food in order to produce water (Lindroth 1993). Relative growth rates of Gypsy moth (*Lymantria dispar*) were reported to be reduced by 30% in larvae fed on *Quercus petraea* exposed to high CO_2_ (Hattenschwiler and Schafellner 2004). However, it is generally believed that CO_2_ induced changes in foliar phytochemical compounds play the most important role in the activities of phytophagous insects. The quantified review of information on published results of the impact of elevated CO_2_ on insect pests through ‘meta analysis’ indicated that the elevated CO_2_ increased relative consumption rate, total consumption, and developmental time, but decreased relative growth rate, conversion efficiency, and pupal weights. Smaller effect sizes for many response variables were reported (Stiling and Corneliessen 2007; Robinson et al. 2012; Srinivasa Rao et al. 2012).

In the present study, an increase in developmental time with decreased pupal weights was observed when larvae of *S. litura* were reared from hatching to pupation on elevated CO_2_ grown peanut crop. A similar observation was reported on cotton leaf worm by Agrell et al. 2006. The fecundity of *S. litura* was found reduced under elevated CO_2_ conditions, and might be due to lower pupal weights. Karowe (1992) observed that pupal weight in insects has a significant and positive correlation with fecundity in Lepidoptera, and a similar trend was noticed in the present study.

A response of an insect herbivore to elevated CO_2_ levels was found to be species-specific, and might occur at different rates, potentially altering growth and development. A complete comprehension of these changes over generations will serve as major input in the development of insect population dynamics models.

## Abbreviation

C: N,: carbon to nitrogen ratio
